# A Case Report of Aberrant Vascular Anatomy of the Anterolateral Thigh Flap

**DOI:** 10.7759/cureus.6385

**Published:** 2019-12-15

**Authors:** S. Hamad Sagheer, William Reschly

**Affiliations:** 1 Otolaryngology, University of Central Florida College of Medicine, Orlando, USA; 2 Otolaryngology, University of Central Florida College of Medicine, Gainesville, USA

**Keywords:** anterolateral thigh flap, anatomy

## Abstract

Soft tissue free flaps are used for a variety of head and neck reconstructions. The anterolateral thigh (ALT) flap has been a versatile tool in head and neck reconstruction since the mainstream use of microvascular anastomosis for free tissue transfer. The ALT flap has a known history of variable vascular anatomy. Most of this variability lies within perforator anatomy and vascular aberrations distal to the lateral circumflex femoral artery (LCF). Few vascular aberrancies have been described proximal to the LCF. Here we present a case of report of an ALT whose arterial vascular pedicle was a branch directly off the femoral artery. The case highlights an unusual anatomical variant of the ALT flap, and the importance of a thorough and meticulous dissection.

## Introduction

The anterolateral thigh (ALT) flap is a pliable flap that is commonly used for reconstruction of large head and neck defects [1]. The versatility of ALT allows it to be raised as a musculocutaneous, fasciocutanous, subcutaneous, or adipofascial flap [1]. Good outcomes with low morbidity of the donor site have been described. For example, division of the nerve to the vastus lateralis may result in gait disturbance, which is specific to the ALT flap [2]. The vascular flap is based on the descending branch of the lateral circumflex femoral (LCF) artery in more than 80% of cases [3]. The pedicle with the perforators often has a diverse anatomical vasculature that can lead to complications in flap reconstruction [4].

## Case presentation

A 65-year-old male was diagnosed with a T3N0M0 (AJCC 8th edition) left oral tongue at the Otolaryngology - Head and Neck Clinic. The multidisciplinary tumor board treatment recommended surgery with postoperative radiation. He was taken to the operating room for near total glossectomy, bilateral selective neck dissections, levels 1-4, and reconstruction with an ALT myocutaneous free flap.

**Figure 1:**
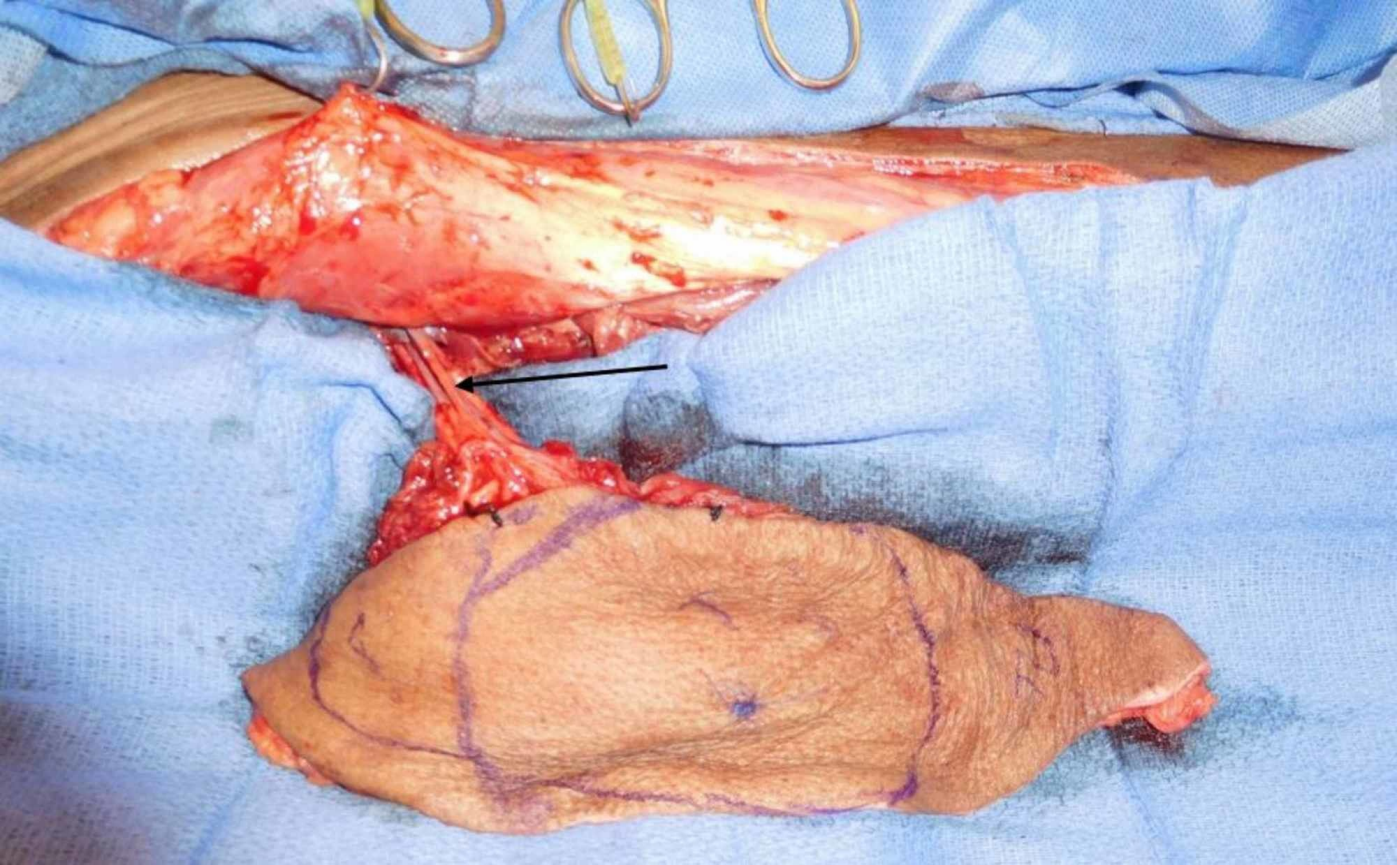
Pedicle to the anterolateral thigh flap directly from the femoral artery

During dissection of the flap, a venous pedicle was found in the anticipated course of the descending branch of the LCF artery. Musculocutaneous perforators were identified. During dissection from distal to proximal, a large caliber artery with associated venae comitantes was found to enter the vastus lateralis. Re-evaluation of the venous pedicle (false pedicle) showed that there was no associated artery. It was determined this aberrant artery was the pedicle to the flap. Retrograde dissection for increased length of the pedicle showed it a direct branch of the femoral artery (Figure 1). The pedicle was proximal to the profunda femoral (PF). The donor site was closed primarily.

Postoperatively the patient adhered well to our institution’s post-free flap protocol. There was good viability of the flap with no wound or flap vascular complications. The donor site healed well with no dysfunction. The patient continues to do well and can swallow a soft mechanical diet. His speech intelligibility has reached 80%.

## Discussion

**Figure 2:**
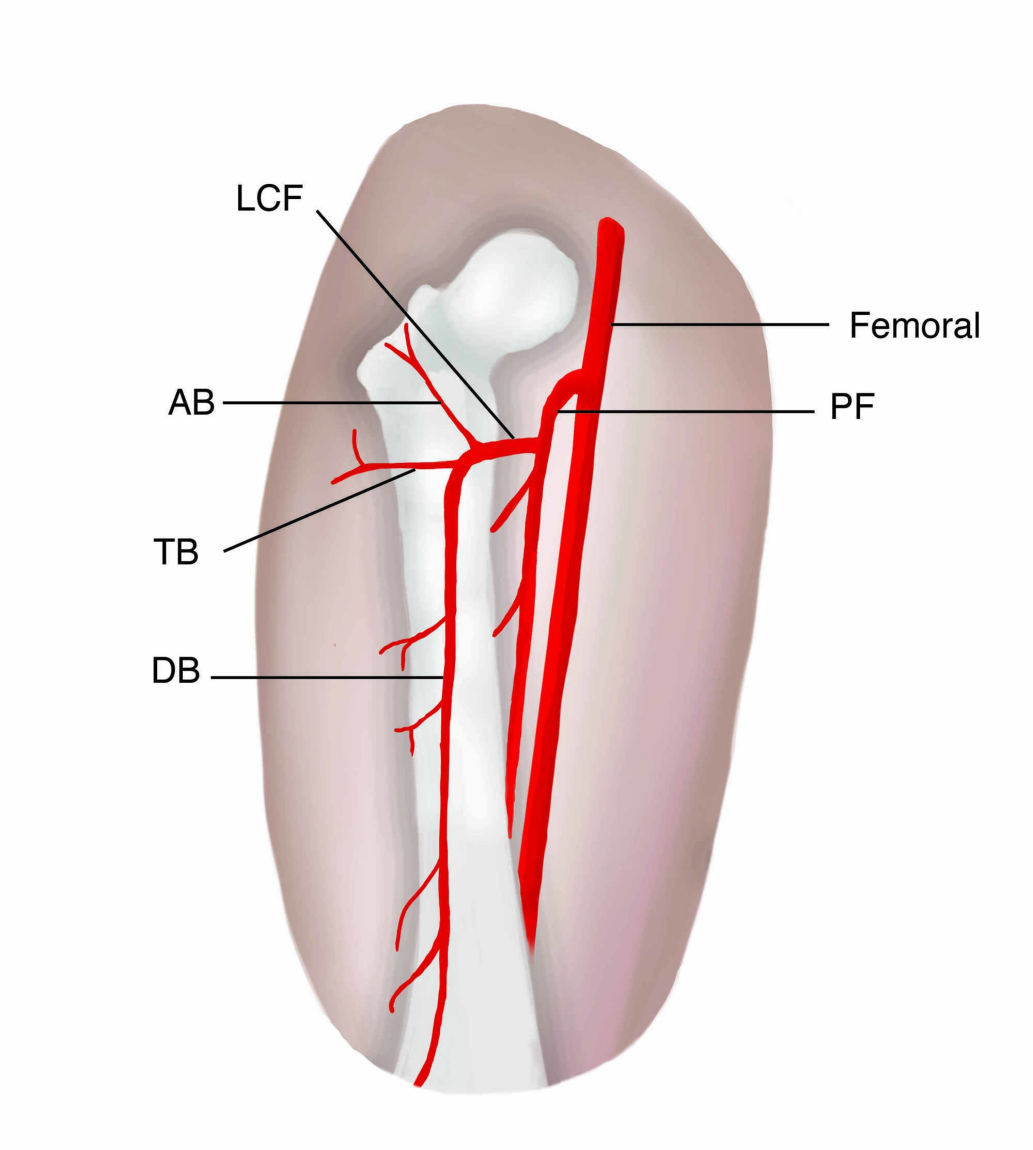
Illustration of normal vascular anatomy of an anterolateral thigh flap LCF, lateral circumflex femoral; PF, profunda femoral; AB, ascending branch; TB, transverse branch; DB, descending branch

The LCF artery system has characteristically been depicted as arising from the deep femoral artery and splitting into ascending, transverse, and descending branches; the latter typically serves as the pedicle for the ALT (Figure 2). Yet, the descending branch has been demonstrated to not be the main artery in as many as 40% of cases [3].

**Figure 3:**
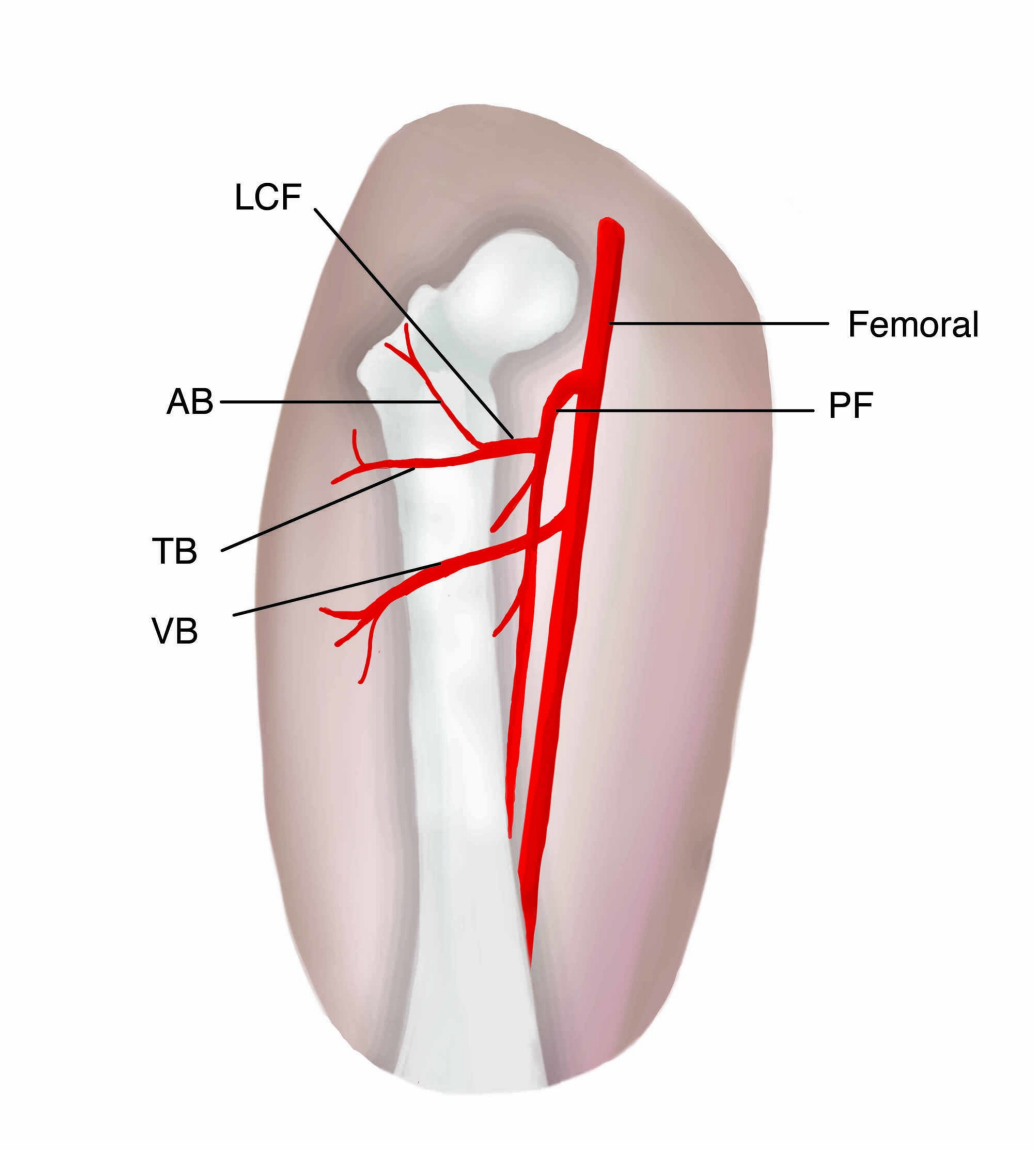
Illustration of the vascular anatomy of the patient LCF, lateral circumflex femoral; PF, profunda femoral; AB, ascending branch; TB, transverse branch; VB, vastus branch

The ALT provides a versatile soft tissue for reconstruction of the head and neck. Minor variances can be expected when utilizing the ALT flap. The case presented a major variance, as the pedicle was not an aberrant branch of the PF or LCF, which are more typical variances (Figure 3). Several papers have described the anatomical variations of the pedicle. For example, one paper described a case of duplication of the descending branch of the LCF artery [5]. In our case, the variance in the vasculature did not seem to impact the vascular flap’s integrity.

Preoperative planning for an ALT flap can help reduce any potential complications during surgery. CT angiography (CTA) could have been utilized but is not a standard preoperative study. A retrospective study in 32 patients compared the surgical result of ALT flaps in cheek defect reconstructions with and without CTA. Usage of preoperative CTA was associated with a significant decrease in major surgical complications and length of surgery [6]. Some studies have explored the use of imaging to depict a flap’s vasculature with limited success [7]. Variations in the vascular anatomy can compromise the length of the exposed pedicle. One study examined the use of preoperative color Doppler sonography with mathematical models to estimate the length of the pedicle, and differentiate anatomical variations. The calculated minimum pedicle length and actual length was congruent in about 75% of cases, and sonography recognized the two anatomical variants of the ALT vasculature in nearly all cases [8]. The experiment reaffirmed the significance of Doppler sonography for ALT flap reconstruction.

## Conclusions

To the best of our knowledge, this is the first case report of an ALT pedicle being a direct branch of the femoral artery. The case highlights a rare anatomical variant and reiterates the significance of a careful dissection. Failure to recognize its variability can lead to flap embarrassment and tissue injury.
